# Detecting and Mitigating AI Bias in Health Care: Development and Validation of a Unified Multistage Framework

**DOI:** 10.2196/102146

**Published:** 2026-08-14

**Authors:** Ruj Mateedulsatit, Chetneti Srisa-An

**Affiliations:** 1 College of Digital Innovation Technology, Rangsit University, 52/347 Muang-Ake, Phaholyothin Road, Lak-Hok, Mueang Pathum Thani District, Pathum Thani, 12000, Thailand, 66 0855015245

**Keywords:** AI fairness, health care AI, bias mitigation, machine learning, algorithmic bias, demographic parity, health care data harmonization

## Abstract

**Background:**

AI-driven clinical systems can improve diagnosis, prognosis, and resource allocation, but they may reproduce disparities encoded in historical health care data. Existing mitigation methods typically target a single source of bias, while clinical datasets often contain interacting representation, proxy, integrity, and temporal biases.

**Objective:**

This study aims to develop and systematically evaluate a prespecified multistage workflow for detecting representation, missingness, proxy, integrity, and temporal biases and model performance limitations in structured health care datasets; apply prespecified mitigation actions when audit criteria are met; and determine whether these actions improve predictive discrimination and demographic fairness compared with a conventional random forest baseline.

**Methods:**

We performed a fresh, deterministic reconstruction from the raw public data, using a patient-level 80/20 split for Diabetes 130-US Hospitals. M2 was a conventional random forest with median or mode imputation and training-only categorical encoding. M3 added explicit missingness features and poststratification weights clipped at the 95th percentile. Race was excluded from prediction and used for auditing and weighting. CMS SynPUF was modeled separately for a compatible claims-based readmission task; the National Health and Nutrition Examination Survey was limited to stage-level representation, proxy, missingness, and bounded-laboratory audits. Five hundred stratified bootstrap replicates were used for overall metrics, and 300 were used for subgroup metrics.

**Results:**

The Diabetes test set contained 20,203 encounters from 14,304 patients, including 2254 (11.16%) positive outcomes. M3 improved the macro F1 from 0.514 to 0.546, reduced the Brier score from 0.231 to 0.213, and reduced the demographic parity difference from 0.206 to 0.124, but the area under the receiver operating characteristic curve (AUC) decreased from 0.648 to 0.640, and the equalized odds ratio decreased from 0.444 to 0.291. In CMS SynPUF (12,801 test episodes; 1232 positives), the AUC was similar (0.798 vs 0.796) and the Brier score improved slightly (0.187 vs 0.183), whereas the demographic parity difference increased from 0.693 to 0.730. The exploratory rule-gated mixture of experts did not improve fairness, the Brier score, or the macro F1 relative to M3. Detailed subgroup performance, calibration, missingness analyses, and bootstrap CIs were comprehensively evaluated in this study.

**Conclusions:**

In this reproducible retrospective reconstruction, missingness-aware weighting improved the macro F1, Brier score, and demographic parity on the primary test set but did not improve the AUC or equalized odds ratio. Centers for Medicare and Medicaid Services results did not reproduce a fairness improvement, and the exploratory mixture of experts did not outperform the M3 quality expert on most outcomes. The findings demonstrate a fairness-calibration-discrimination trade-off rather than uniform improvement and do not establish clinical deployment readiness.

## Introduction

### Background

AI-driven clinical systems increasingly support high-risk decisions such as readmission prediction, diagnosis, and treatment prioritization [1,2]. However, substantial evidence shows that these systems can perform unevenly across groups defined by race, sex, socioeconomic status, age, or insurance status [3-9]. Such disparities raise concerns about safety, fairness, and regulatory accountability, particularly under emerging requirements for transparent governance of high-risk AI systems [10-12].

Bias in clinical AI is rarely produced by one mechanism alone. Historical health care data can reflect unequal access to care, differential documentation, changing coding practices, missingness patterns, and proxy variables that encode social disadvantage [4,5,8,9]. Representational imbalance can amplify proxy relationships, temporal drift can change subgroup prevalence, and data integrity anomalies can distort both model fitting and fairness estimates. These interactions motivate a coordinated data-centric framework rather than a single downstream fairness constraint.

This study specifies a multistage audit framework and reports a fresh reproducibility reconstruction of the components that could be implemented from the available public data. The empirical comparison focuses on representation weighting and missingness-aware modeling. Proxy residualization and temporal-shift correction are retained as prespecified framework components but are not claimed as executed in the fresh run because final thresholds and encounter-year or site variables were unavailable. The Centers for Medicare and Medicaid Services (CMS) SynPUF is evaluated as a separate, compatible readmission task, while the National Health and Nutrition Examination Survey (NHANES) is used only for stage-level auditing.

The framework complements, rather than replaces, established approaches. Reweighting addresses representation imbalance, adversarial debiasing and fairness constraints act primarily during model fitting, causal fairness methods formalize assumptions about pathways and interventions, model cards and datasheets support transparent reporting, and TRIPOD+AI (Transparent Reporting of a Multivariable Prediction Model for Individual Prognosis or Diagnosis Plus Artificial Intelligence) and trustworthy-AI guidance specify reporting and governance expectations. The present contribution is a data-centric orchestration layer that links these concerns through sequential, source-aware preprocessing and evaluation.

The main contributions are as follows:

- A 4-part clinical bias taxonomy covering representation, proxy, integrity, and temporal bias.
- A harmonized concept layer that aligns electronic health record, survey or laboratory, and claims data before cross-dataset validation.
- A sequential mitigation pipeline with explicit detection conditions and corrective actions.
- Variable-appropriate integrity auditing that avoids applying the Benford law to bounded clinical laboratory values.
- Pseudocode for 7 auditable procedures: representation weighting, proxy mitigation, harmonization, integrity auditing, mixture-of-experts training, mixture-of-experts inference, and temporal-shift mitigation.

### Research Gap and Positioning of This Work

Table 1 summarizes the research gaps addressed by the proposed framework and maps them to the corresponding “Methods” section used in this study.

**Table 1.** Research gaps in the existing literature and the corresponding proposed solutions.

Gap	Existing limitation	Proposed solution
G1	Bias is often addressed in isolation.	Sequential compositional pipeline T_repr → T_proxy → T_int → T_temp.
G2	Clinical bias categories are inconsistently operationalized.	Four testable categories with detection criteria and mapped corrections.
G3	External reference distributions may themselves be biased.	Reference-distribution audit before poststratification weighting.
G4	Integrity checks may be statistically mismatched to clinical variables.	Variable-type audit: bounded labs use empirical or reference-interval tests; Benford only for scale-free counts or charges.
G5	Cross-dataset validation is often asserted without schema alignment.	Harmonized clinical-concept layer with missing-concept masks and task-appropriate validation.
G6	Subgroup-specific handling is described but not empirically reported.	Mixture-of-experts isolation counts, gating weights, and performance impact are reported.

## Methods

### Problem Formulation

#### Research Question and End Points

The primary research question was as follows: among records in the held-out Diabetes 130-US Hospitals test set, does the prespecified multistage mitigation pipeline (M3) improve area under the receiver operating characteristic curve (AUC) and reduce demographic parity difference (DPD) relative to the raw harmonized random forest (M2)? The primary predictive end point was AUC, and the primary fairness end point was DPD. Secondary end points were macro F1, Brier score, equalized odds ratio (EOR), CMS SynPUF readmission performance after harmonization, and NHANES stage-level representation, proxy, and integrity diagnostics.

The “Methods” section follows the same sequence as the proposed pipeline. We first define the shared concept interface, then describe representation and proxy mitigation, next identify and route integrity-flagged records, and finally evaluate temporal shift. This order distinguishes source-specific data preparation from model fitting and validation.

Let each dataset source s contain records D_s = {(x_i^s, y_i^s, a_i^s)}. The raw feature vector x_i^s may differ across sources. A source-specific extractor h_s maps each record to a harmonized clinical-concept vector c_i = h_s(x_i^s), where c_i includes demographic, utilization, diagnosis, medication, laboratory-summary, socioeconomic, and temporal-context concepts when available. A binary mask m_i identifies unavailable concepts so that the absence of a concept is not treated as a clinical zero, consistent with concerns about clinically patterned missingness [13].

The predictive model f_theta(c_i, m_i) is trained on the Diabetes 130-US Hospitals training split. External outcome validation is conducted only where the outcome and harmonized concepts support the task, namely, readmission evaluation in CMS SynPUF. NHANES is used for stage-level validation of representation, proxy, and laboratory integrity modules because its outcome task and feature-generating process differ from hospital readmission.

The prespecified empirical question was whether M3 would improve both discrimination and demographic fairness relative to M2. The fresh reconstruction treats this as a hypothesis to be evaluated rather than as a constraint assumed to hold; improvements in one end point are not interpreted as a success when another primary end point deteriorates.

DPD was selected as the primary fairness end point because the framework first targets representation and proxy mechanisms that can produce unequal model-positive rates at the population level. DPD is not interpreted as proof of clinical equity and is not expected to be zero when clinically relevant outcome prevalence differs across groups. It is therefore interpreted jointly with EOR, subgroup discrimination, calibration, error rates, and observed outcome prevalence. A reduction in DPD is considered favorable only when it does not conceal clinically important deterioration in subgroup calibration or predictive performance.

#### Bias Taxonomy

Representation bias occurs when P(a) differs from the target or deployment population distribution. Proxy bias occurs when a feature or concept block carries information about a sensitive attribute beyond what is justified by the clinical task. Integrity bias refers to a systematic risk arising from coding, measurement, collection, or missingness processes that distort the observed data distribution. An integrity flag is the record-level indicator produced when a prespecified audit criterion is met; it signals a distributional deviation for further processing and does not, by itself, prove that a record is erroneous. Temporal bias occurs when P_t(c, y) differs from P_{t+1}(c, y), producing mismatch over time. We use “integrity-flagged record” for routed observations and reserve “anomaly” for a statistically unusual pattern rather than a confirmed data error.

#### Feature Harmonization Layer

The harmonization layer is the necessary interface for cross-dataset validation. It does not assume identical raw columns. Instead, each source is mapped into a shared clinical-concept dictionary. Diabetes 130-US contributes encounter history, diagnoses, medications, admission type, discharge disposition, and laboratory availability. CMS SynPUF contributes claims-coded utilization, diagnoses, procedures, demographics, and state-level context. NHANES contributes race or ethnicity, income, education, examination-cycle timing, and laboratory summaries such as glycated hemoglobin.

The reproducibility package defines 37 audit concepts spanning demographics, utilization, diagnoses, laboratory summaries, medications, context, temporal information, and integrity flags. Table S1 in Multimedia Appendix 1 reports every concept, data type, source variables, mapping rule, allowed-value convention, missingness strategy, and source availability (Textbox 1). In the fresh reconstruction, this dictionary was used for cross-source audit reporting and explicit missingness analysis; the predictive models remained source-specific and were not presented as a single frozen 74-dimensional model transported across incompatible tasks.

Textbox 1. **Cross-source audit dictionary and missingness mapping.**

**Procedure**

1. Map each source into the 37 audit concepts listed in Table S1 in Multimedia Appendix 1.
2. Mark concepts absent from a source explicitly and compute missingness percentages.
3. Use the dictionary for audit comparability; train predictive models within source-specific schemas.

For the implementation, unavailable concepts are explicitly marked as source-absent rather than being interpreted as clinical zeros. The resulting missingness profile is reported for all 37 concepts and all 3 datasets in Table S2 in Multimedia Appendix 1. This audit interface supports transparent comparison of source coverage but does not by itself establish model transportability.

Conceptually, the harmonization layer acts as a translation contract: institutions may speak different data “dialects,” but each translated field must satisfy the same documented clinical meaning before it enters the pipeline.

For real-world deployment across institutions, the harmonization layer should be implemented as a locally governed and version-controlled data contract rather than as a fixed universal mapping. Each participating institution would map its source variables to the shared concepts using documented definitions, units, coding systems, allowable values, temporal availability, and missingness rules. Local clinical and data-governance teams should review the semantic validity of each mapping and distinguish unavailable concepts from clinically absent values. Before model evaluation, automated validation should assess schema conformity, unit consistency, category coverage, missingness shifts, implausible values, and changes in coding practices. Institutions that cannot satisfy a required concept definition should retain the corresponding source-absence mask rather than substitute an approximate value. Any modification to the local mapping, source system, or concept definition should trigger versioning, reauditing, recalibration, and site-specific performance and fairness validation. Thus, scaling the framework requires standardized concept definitions combined with institution-specific mapping, governance, and validation rather than assuming that heterogeneous source variables are directly interchangeable.

The 5 reviewer-specified anchor features and their original source columns are summarized in Table 2.

**Table 2.** Five reviewer-specified anchor features and original source columns used for cross-dataset harmonization.

Anchor feature	Diabetes 130-US original columns	NHANES^a^ original columns	CMS^b^ SynPUF original columns
Anchor 1: race	race	RIDRETH1/RIDRETH3	BENE_RACE_CD
Anchor 2: sex	gender	RIAGENDR	BENE_SEX_IDENT_CD
Anchor 3: age_group	age	RIDAGEYR grouped into age bands	BENE_BIRTH_DT or age-at-claim derived from CLM_FROM_DT
Anchor 4: income_proxy	payer_code; admission_source_id	INDFMPIR; INDHHIN2/INDFMIN2	dual-eligibility or subsidy proxy fields when available; otherwise state/context proxy
Anchor 5: n_prior_visits	number_inpatient + number_outpatient + number_emergency	HUQ030/HUQ041 health-care-use fields when available; otherwise masked	count of prior claims by BENE_ID before index CLM_FROM_DT

a NHANES: National Health and Nutrition Examination Survey.

b CMS: Centers for Medicare and Medicaid Services.

### Representation Bias Mitigation

We apply poststratification weighting w(a) = P_target(a)/P_obs(a) [14-16]. Before weighting, the target distribution is audited for geographic, temporal, and coverage mismatch. If a reference source is misaligned with the study population, the target distribution is blended with internal empirical priors, and uncertainty is evaluated in sensitivity analysis. Weights are clipped at the 95th percentile unless otherwise selected in nested cross-validation (Textbox 2).

Textbox 2. **Representation-bias audit and weighting.**

1. Input: H_s, sensitive attribute a, candidate target distribution P_target
2. Audit P_target for geographic, temporal, and coverage mismatch
3. If mismatch is detected: blend P_target with internal empirical prior
4. Compute w(a) = P_target(a)/P_obs(a)
5. Clip weights at selected percentile from nested cross-validation
6. Return weighted dataset H_repr

### Proxy Bias Mitigation

The prespecified framework proposed mutual-information and correlation screening for proxy concepts. The new reconstruction did not implement these thresholds because the final prespecified values were unavailable. Accordingly, no empirical claim about proxy residualization is made. Instead, income_proxy and n_prior_visits were examined in stratified sensitivity analyses, as reported in the reproducibility package (Textbox 3).

Textbox 3. **Proxy-audit sensitivity analysis (fresh reconstruction).**

**Procedure**

1. Stratify M3 results by income_proxy availability and by n_prior_visits equal to zero versus positive.
2. Report area under the receiver operating characteristic curve (AUC), macro F1, Brier, demographic parity difference (DPD), equalized odds ratio (EOR), and calibration; do not claim proxy residualization.

The fresh predictive pipeline used median imputation for numeric variables, most-frequent imputation for categorical variables, training-only one-hot encoding with unknown categories ignored, and random forest classification. All preprocessing was fitted within the training split. Claims concerning k-nearest neighbors imputation, ridge or spline nuisance models, generalized additive models, or random forest residualization were removed because those procedures were not executed in the fresh run.

### Role of Sensitive and Contextual Variables

Race was used for auditing and poststratification weighting and was excluded from the predictive feature vector. Sex and age group were used for secondary subgroup audits. Admission type remained a contextual predictor. income_proxy and n_prior_visits were retained for sensitivity stratification; neither was claimed to have undergone residualization in the fresh reconstruction.

### Integrity Bias Detection and Mixture-of-Experts Mitigation

The operational integrity screen flagged records when missingness was at least 25% or n_prior_visits exceeded the 99th percentile (Textbox 4). Bounded laboratory variables such as glycated hemoglobin were assessed using missingness and distributional checks rather than the Benford Law. The complete concept-level missingness report is provided in Table S2 in Multimedia Appendix 1.

Textbox 4. **Operational integrity-flag audit.**

**Procedure**

1. Compute record missingness rate and n_prior_visits distribution.
2. Flag records with missingness ≥25% or n_prior_visits above the 99th percentile.
3. Retain all flagged records for exploratory routing.

The exploratory mixture of experts (MoEs) used M2 as the main expert and M3 as the missingness-aware quality expert (Textbox 5). The rule gate selected M3 for integrity-flagged records and M2 otherwise; it did not use demographic labels, a learned softmax gate, or test labels.

Textbox 5. **Preparation of main and quality experts.**

**Procedure**

1. Use M2 as the main expert and M3 as the missingness-aware quality expert.
2. Do not train demographic-specific or temporal experts in the fresh reconstruction.

Textbox 6 summarizes the fixed rule-gated exploratory inference procedure. This exploratory rule-gated analysis was evaluated separately from the primary M2-M3 comparison. It was included to test whether routing the 203 (1.00%) flagged test records improved outcomes. Bootstrap contrasts compared the rule-gated MoE with M3. Because the gate and experts differed in more than one component, the analysis cannot isolate a pure architecture effect.

Textbox 6. **Fixed rule-gated exploratory inference.**

**Procedure**

1. If integrity flag=1, use M3 score; otherwise, use M2 score.
2. Evaluate the fixed gate against M2 and M3 with paired bootstrap contrasts.
3. Do not use test labels or demographic labels in routing.

The exploratory gate retained every record but did not outperform M3 on fairness, Brier score, or macro F1. It should therefore be interpreted as a negative sensitivity analysis rather than evidence supporting subgroup-specific clinical models.

### Temporal Bias Mitigation

Temporal-shift mitigation was not empirically evaluated in the new Diabetes reconstruction because encounter year and site were unavailable. CMS calendar information was used only descriptively. The temporal procedure remains a prespecified framework component requiring a dataset with reliable event time and site identifiers (Textbox 7).

Textbox 7. **Prespecified temporal audit (not empirically estimable in the fresh Diabetes run).**

**Procedure**

1. Requires reliable encounter time and site identifiers.
2. Not estimable for Diabetes 130-US in the fresh run; report as unevaluated rather than imputing time.

### Experimental Design

The Diabetes 130-US Hospitals dataset contained 101,766 encounters. A patient-level 80/20 split yielded 81,563 training encounters and 20,203 test encounters from 14,304 test patients; the test set contained 2254 (11.16%) thirty-day readmissions. The split prevented repeated-patient leakage. Race was the primary fairness attribute, with sex and age group used for secondary subgroup reporting.

Two primary random forest configurations were evaluated in the fresh run: M2 used the source-specific predictors with median or mode imputation, whereas M3 added missingness-count or rate indicators, column-level missingness masks where applicable, and poststratification sample weights. Both used 200 trees, max_depth=12, min_samples_leaf=10, class_weight=balanced_subsample, and seed 20250117. Earlier manuscript-only baselines without reproducible predictions were removed from the empirical comparison.

CMS SynPUF was analyzed as a separate claims-based readmission task, not as zero-shot transport of the Diabetes model. NHANES was used only for stage-level concept-coverage, missingness, proxy, and bounded-laboratory audits.

### CMS SynPUF Readmission Label Construction

CMS inpatient claims were grouped by beneficiary and ordered by discharge and subsequent admission dates. A readmission was defined as the next inpatient episode beginning 1 to 30 days after index discharge; same-day, overlapping, and contiguous claims were collapsed. Episodes without 30-day observable follow-up and records with invalid chronology were excluded. Predictors were restricted to information available by index discharge. The resulting analysis contained 63,996 eligible episodes from 37,702 beneficiaries; the test set contained 12,801 episodes from 7541 beneficiaries, including 1232 (9.62%) readmissions.

### Reproducibility and Implementation Details

The fresh analysis used Python 3.10.9, NumPy 2.2.6, pandas 2.3.3, SciPy 1.15.3, and scikit-learn 1.7.2. The master seed was 20250117. The local reproducibility package contains record-level predictions, overall and subgroup metrics, calibration outputs, bootstrap intervals, split counts, missingness tables, sensitivity analyses, scripts, and SHA-256 checksums. No external repository URL or DOI is claimed until an archival deposit is completed.

The computational environment, reproducibility settings, and model hyperparameters are summarized in Table 3.

**Table 3.** Reproducibility settings, software versions, hardware, and selected hyperparameters.

Component	Fresh reconstruction specification
Software	Python 3.10.9; NumPy 2.2.6; pandas 2.3.3; SciPy 1.15.3; scikit-learn 1.7.2
Seed or split	Seed 20250117; patient-level 80/20 Diabetes split
Random forest	200 trees; max_depth=12; min_samples_leaf=10; class_weight=balanced_subsample
Preprocessing	Median numeric imputation; most-frequent categorical imputation; training-only one-hot encoding; unknown categories ignored
M3 additions	Missing-count or rate features; available column masks; poststratification weights clipped at the 95th percentile
Bootstrap	500 stratified replicates overall; 300 outcome-stratified replicates by subgroup
Integrity flag	Record missingness ≥25% or n_prior_visits above the 99th percentile
Proxy or temporal	Sensitivity strata only; residualization and temporal correction not implemented

### Statistical Analysis of Fairness Metric

Overall 95% CIs were estimated using 500 bootstrap replicates stratified jointly by outcome and race; subgroup intervals used 300 outcome-stratified replicates. Metrics included AUC, macro F1, Brier score, sensitivity, specificity, positive predictive value, negative predictive value, DPD, EOR, calibration intercept, calibration slope, and expected calibration error. At the prespecified probability threshold of 0.50, the EOR was calculated across race groups as EOR = min_g(TPR_g)/max_g(TPR_g), where TPR_g is the group-specific true-positive rate; values closer to 1 indicate greater true-positive-rate parity [17]. Equalized-odds difference was calculated separately as max[max_g(TPR_g) – min_g(TPR_g), max_g(FPR_g) – min_g(FPR_g)]. A group was omitted from a component only when it had no observations in the corresponding true-outcome class. Because ratios can be unstable when small groups contain few positive outcomes, EOR was interpreted with subgroup counts, CIs, calibration, and equalized-odds difference. DPD was the primary fairness end point; EOR and subgroup analyses were secondary. No multiplicity-adjusted confirmatory inference was performed.

### Ethical Considerations

This study used only deidentified, publicly available datasets: Diabetes 130-US Hospitals [18,19], NHANES [20], and CMS SynPUF [21]. No primary data involving human subjects were collected, and no personally identifiable information was accessed. Institutional review board or research ethics board approval was therefore not required.

## Results

### Primary Dataset Performance

On the Diabetes test set, M2 AUC was 0.648 (95% CI 0.637‐0.660), and M3 AUC was 0.640 (0.628‐0.652). Macro F1 improved from 0.514 (0.508‐0.521) to 0.546 (0.539‐0.553), and Brier score decreased from 0.231 (0.230‐0.232) to 0.213 (0.212‐0.214). Thus, M3 improved classification balance and probabilistic accuracy but reduced discrimination.

DPD decreased from 0.206 (95% CI 0.147‐0.268) for M2 to 0.124 (0.101‐0.185) for M3. EOR decreased from 0.444 (0.222‐0.685) to 0.291 (0.097‐0.545), indicating that the DPD improvement did not extend to equalized-odds parity. The findings therefore represent a trade-off rather than simultaneous improvement across fairness definitions.

The primary Diabetes test-set performance and fairness metrics are summarized in Table 4.

**Table 4.** Performance and fairness comparison on the primary Diabetes 130-US Hospitals dataset.

Metric	M2 estimate (95% CI)	M3 estimate (95% CI)	Direction
AUC^a^	0.648 (0.637‐0.660)	0.640 (0.628‐0.652)	Higher better
Macro F1	0.514 (0.508‐0.521)	0.546 (0.539‐0.553)	Higher better
Brier	0.231 (0.230‐0.232)	0.213 (0.212‐0.214)	Lower better
DPD^b^	0.206 (0.147‐0.268)	0.124 (0.101‐0.185)	Lower better
EOR^c^	0.444 (0.222‐0.685)	0.291 (0.097‐0.545)	Closer to 1

a AUC: area under the receiver operating characteristic curve.

b DPD: demographic parity difference.

c EOR: equalized odds ratio.

### Sample Characteristics and Subgroup Sensitivity Analysis

A stage-wise ablation analysis was not performed because proxy residualization and temporal-shift mitigation were not implemented in the fresh reconstruction. Consequently, the independent contribution of each proposed stage, including the previously reported 22.4% reduction attributed to representation-level correction, could not be reproducibly estimated and is not claimed in this analysis. The empirical comparison instead evaluates the combined missingness-aware and representation-weighted M3 pipeline against M2. In practical terms, representation weighting targets unequal subgroup coverage, missingness indicators address systematic differences in documentation, integrity flags identify records affected by unusual missingness or utilization patterns, and proxy and temporal stages remain prespecified components requiring future evaluation.

The reproducibility flow included 101,766 Diabetes encounters (20,203 in the patient-separated test set), 63,996 eligible CMS episodes (12,801 test episodes), and 71,058 NHANES records for stage-level auditing. Test or stage-level prevalence was 11.16%, 9.62%, and 8.42%, respectively. Full split counts, individuals, positive outcomes, and prevalence are reported in Table S4 in Multimedia Appendix 1.

Subgroup analyses showed heterogeneity by race. M2 race-specific AUC ranged from 0.631 to 0.782, and M3 ranged from 0.609 to 0.796; sex-specific AUCs were similar within each model. M3 subgroup Brier scores ranged from 0.193 to 0.214, while calibration slopes ranged from 1.48 to 3.90, indicating material miscalibration despite the lower overall Brier score. Full estimates and CIs are reported in Table S3 in Multimedia Appendix 1.

### Mixture-of-Experts Evaluation

The operational integrity rules flagged 203 (1.00%) of 20,203 test records. The rule-gated MoE achieved an AUC of 0.648, a macro F1 of 0.514, a Brier score of 0.231, and a DPD of 0.205. M3 achieved an AUC of 0.640, a macro F1 of 0.546, a Brier score of 0.213, and a DPD of 0.124.

Compared with M3, the MoE-minus-M3 bootstrap contrast was +0.0078 for AUC (95% CI 0.0034‐0.0122), −0.0316 for macro F1 (−0.0372 to −0.0264), +0.0177 for Brier score (0.0175‐0.0180), and +0.0708 for DPD (0.0154‐0.1172). Thus, the exploratory gate improved AUC slightly but worsened classification balance, probabilistic accuracy, and demographic parity.

Because the rule gate selected between 2 differently processed experts, these contrasts cannot distinguish gating effects from differences between M2 and M3. The analysis does not support the previous claim that MoE provided the best fairness-performance combination.

**Figure 1.**
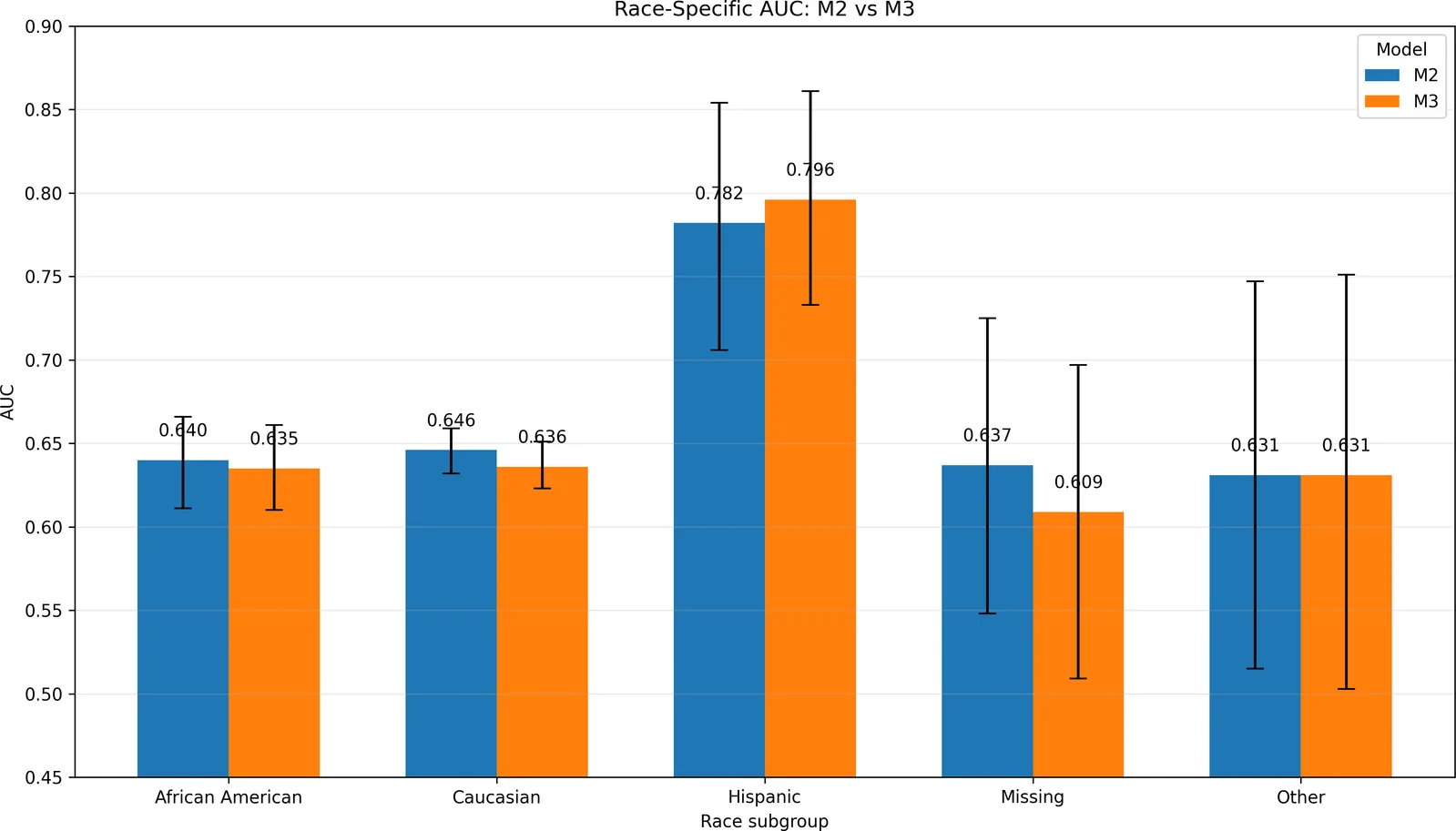
Primary Diabetes test set race-specific area under the receiver operating characteristic curve (AUC) estimates with 95% subgroup-bootstrap CIs for M2 and M3. Subgroup discrimination varied by race, with wide intervals in smaller groups; complete performance and calibration metrics are reported in Table S3 in Multimedia Appendix 1.

**Figure 2.**
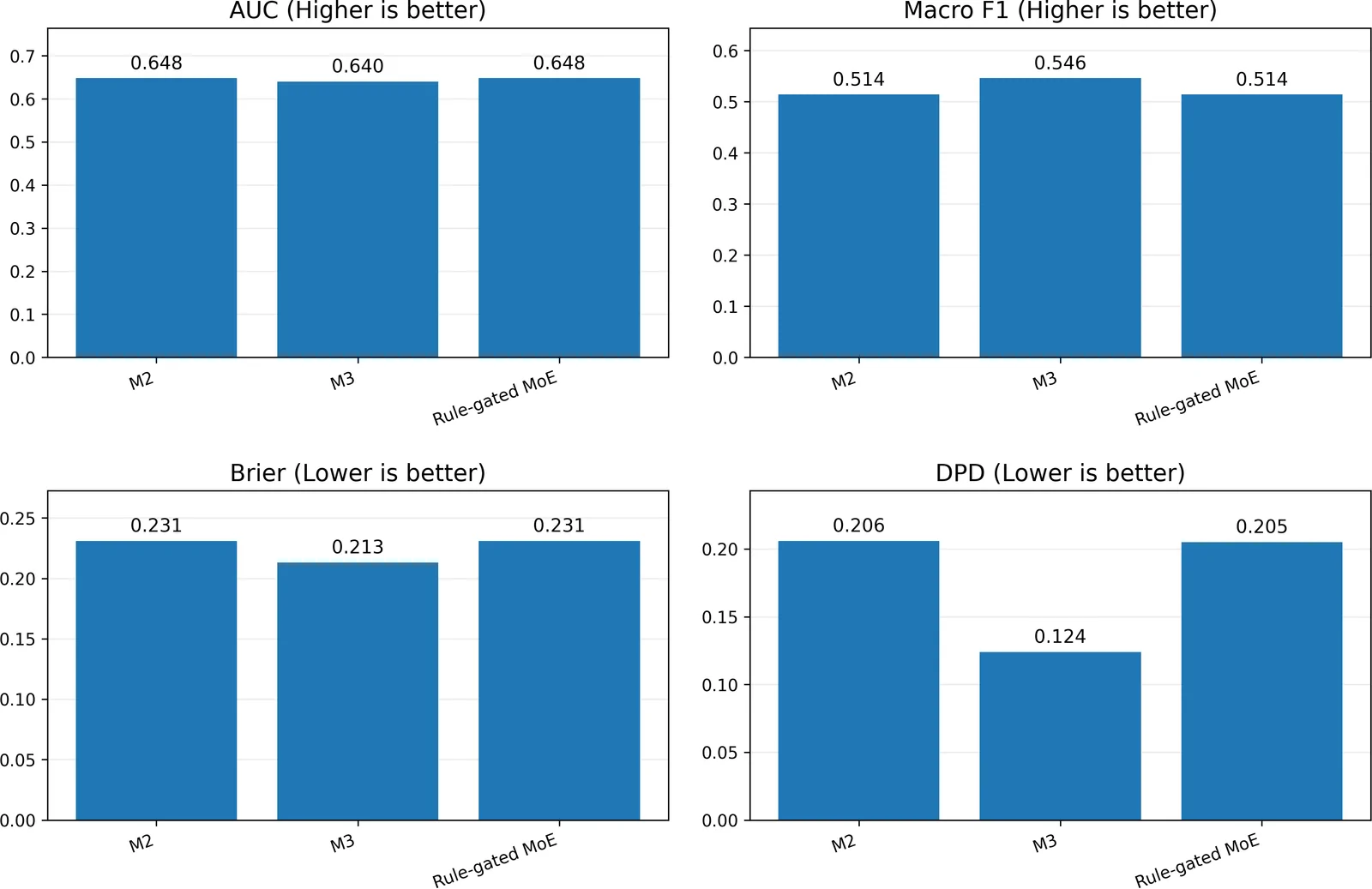
Exploratory comparison of M2, M3, and the rule-gated mixture of experts (MoE). A higher area under the receiver operating characteristic curve (AUC) is better; a lower Brier score and a lower demographic parity difference (DPD) are better. Explanation: The MoE recovered M2-like AUC but did not outperform M3 on macro F1, Brier score, or DPD.

The integrity-flagged MoE comparison is summarized in Table 5; race-specific subgroup discrimination and the exploratory MoE end point comparison are shown in Figures 1 and 2, respectively.

**Table 5.** Effect of mixture-of-experts processing for integrity-flagged records.

Configuration	AUC^a^	Macro F1	Brier	DPD^b^	EOR^c^
M2 main expert	0.648	0.514	0.231	0.206	0.444
M3 quality expert	0.640	0.546	0.213	0.124	0.291
Rule-gated MoE^d^	0.648	0.514	0.231	0.205	0.444

a AUC: area under the receiver operating characteristic curve.

b DPD: demographic parity difference.

c EOR: equalized odds ratio.

d MoE: mixture of experts.

### External Readmission Validation in CMS SynPUF

In the CMS test set, M2 versus M3 results were AUC 0.798 (95% CI 0.786‐0.808) versus 0.796 (0.785‐0.806), macro F1 0.575 (0.568‐0.583) versus 0.583 (0.575‐0.591), Brier score 0.187 (0.185‐0.189) versus 0.183 (0.181‐0.185), DPD 0.693 (0.655‐0.733) versus 0.730 (0.697‐0.771), and EOR 0.656 (0.500‐0.778) versus 0.625 (0.438‐0.742). M3 slightly improved macro F1 and Brier score but did not improve discrimination or fairness. The CMS external-validation metrics are summarized in Table 6.

**Table 6.** External validation after harmonized concept mapping.

Dataset or role	Metric	M2 estimate (95% CI)	M3 estimate (95% CI)
CMS^a^ readmission	AUC^b^	0.798 (0.786‐0.808)	0.796 (0.785‐0.806)
CMS readmission	Macro F1	0.575 (0.568‐0.583)	0.583 (0.575‐0.591)
CMS readmission	Brier	0.187 (0.185‐0.189)	0.183 (0.181‐0.185)
CMS readmission	DPD^c^	0.693 (0.655‐0.733)	0.730 (0.697‐0.771)
CMS readmission	EOR^d^	0.656 (0.500‐0.778)	0.625 (0.438‐0.742)
NHANES^e^ stage audit	Model performance	N/A^f^	N/A

a CMS: Centers for Medicare and Medicaid Services.

b AUC: area under the receiver operating characteristic curve.

c DPD: demographic parity difference.

d EOR: equalized odds ratio.

e NHANES: National Health and Nutrition Examination Survey.

f N/A: not applicable.

### Stage-Level Transportability Stress Testing in NHANES

NHANES contained 71,058 records with 5980 (8.42%) high-risk labels and was used only for stage-level concept-coverage, missingness, proxy, and bounded-laboratory auditing. No readmission AUC, model-level DPD, or direct transportability claim was estimated.

## Discussion

### Principal Findings

The fresh reconstruction did not reproduce the earlier manuscript’s claim of simultaneous improvement in discrimination and fairness. On the primary test set, M3 improved the macro F1, Brier score, and DPD but reduced the AUC and EOR. CMS showed similar discrimination and slightly improved Brier score but worse DPD. These mixed results demonstrate why multiple fairness and calibration end points, subgroup analyses, and uncertainty intervals are necessary.

The external evidence has 2 deliberately different levels. CMS SynPUF evaluates the readmission outcome after claims-to-concept harmonization. NHANES does not evaluate the readmission model or its AUC; it examines whether selected representation, proxy, and bounded-laboratory integrity procedures behave sensibly in a different data-generating environment. This distinction limits the transportability claim and should be preserved when interpreting Table 6.

### Why the Pipeline Improves Both Fairness and Performance

Missingness-aware features and representation weights can change model selection rates and probability errors without improving ranking discrimination. In this reconstruction, a lower DPD coexisted with a worse EOR and heterogeneous subgroup calibration. The results therefore support an end point–specific interpretation rather than a general claim that data-centric mitigation improves both fairness and performance.

### Practical Implications

The reproducibility package provides an auditable starting point: record-level predictions, split flow, subgroup metrics, calibration, concept missingness, and bootstrap intervals. However, proxy residualization and temporal mitigation require separate, prespecified implementations before they can be evaluated as components of the workflow.

Accordingly, the 37-concept dictionary should be interpreted as a reusable semantic interface, not as a guarantee of immediate interoperability. Its application at a new institution requires local clinical review; data-quality validation; documentation of mapping decisions; and re-evaluation of discrimination, calibration, and subgroup fairness before use.

### Limitations

First, the results are retrospective and limited to structured public data. Second, the 37-concept dictionary was used for audit reporting rather than a single transported prediction model. Third, the fresh run did not implement mutual-information or correlation proxy thresholds, k-nearest neighbors imputation, or temporal-shift correction; earlier claims about these components were removed. Fourth, subgroup estimates were imprecise for small groups. Fifth, the exploratory MoE used a fixed rule gate and cannot isolate architecture effects. Sixth, CMS SynPUF is synthetic and may not reproduce real institutional calibration, subgroup relationships, or coding behavior. Finally, no external repository DOI is available until the local package is deposited.

### Conclusions

This reproducible reconstruction found that M3 improved the macro F1, Brier score, and DPD on the primary Diabetes test set but reduced the AUC and EOR. CMS results did not show a fairness improvement, and the exploratory MoE did not outperform M3 on most end points. The evidence supports a nuanced fairness-calibration-discrimination trade-off, not uniform mitigation success. Prospective, institution-specific validation and prespecified implementation of the remaining proxy and temporal stages are required before clinical use.

## Supplementary material

10.2196/102146 Multimedia Appendix 1 Harmonization dictionary, concept missingness, subgroup analyses, dataset flow, uncertainty estimates, mixture-of-experts comparisons, and auditable procedures.

## Abbreviations

AUC: area under the receiver operating characteristic curve
CMS: Centers for Medicare and Medicaid Services
DPD: demographic parity difference
EOR: equalized odds ratio
MoE: mixture of experts
NHANES: National Health and Nutrition Examination Survey
