# Correction: Detecting and Mitigating AI Bias in Health Care: Development and Validation of a Unified Multistage Framework

**DOI:** 10.2196/114624

**Published:** 2026-11-04

**Authors:** Ruj Mateedulsatit, Chetneti Srisa-An

**Affiliations:** 1 College of Digital Innovation Technology, Rangsit University, 52/347 Muang-Ake, Phaholyothin Road, Lak-Hok, Mueang Pathum Thani District, Pathum Thani, 12000, Thailand, 66 0855015245

In “Detecting and Mitigating AI Bias in Health Care: Development and Validation of a Unified Multistage Framework” [1], the authors noted two errors.

First, the Statistical Analysis section contains a paragraph that duplicates the complete Statistical Analysis paragraph immediately preceding it and includes the corrupted text “replic Metrics.” The following paragraph was removed:

*Overall were estimated using 500 bootstrap replicates stratified jointly by outcome and race; subgroup intervals used 300 outcome-stratified replic Metrics included AUC, macro F1, Brier score, sensitivity, specificity, positive predictive value, negative predictive value, DPD, EOR, calibration intercept, calibration slope, and expected calibration error. At the prespecified probability threshold of 0.50, the EOR was calculated across race groups as EOR = min_g(TPR_g)/max_g(TPR_g), where TPR_g is the group-specific true-positive rate; values closer to 1 indicate greater true-positive-rate parity[17]. Equalized-odds difference was calculated separately as max[max_g(TPR_g) – min_g(TPR_g), max_g(FPR_g) – min_g(FPR_g)]. A group was omitted from a component only when it had no observations in the corresponding true-outcome class. Because ratios can be unstable when small groups contain few positive outcomes, EOR was interpreted with subgroup counts, CIs, calibration, and equalized-odds difference. DPD was the primary fairness end point; EOR and subgroup analyses were secondary. No multiplicity-adjusted confirmatory inference was performed.*

The citation of reference 17 has also been moved into the remaining text as follows:

values closer to 1 indicate greater true-positive-rate parity [17].

In the Sample Characteristics and Subgroup Sensitivity Analysis section, the following paragraph has been removed:

*The evaluation sample sizes across the Diabetes, CMS, and NHANES analyses were as follows: n=20,354, n=12,732, and n=45,565, respectively. The observed outcome or high-risk prevalences across the Diabetes, CMS, and NHANES analyses were as follows: 11.2%, 9.4%, and 8.8%, respectively.*

The correct verified values are already reported in an earlier paragraph.

The correction will appear in the online version of the paper on the JMIR Publications website together with the publication of this correction notice. Because this was made after submission to PubMed, PubMed Central, and other full-text repositories, the corrected article has also been resubmitted to those repositories.
