# Supplementary material for: Detecting and Mitigating AI Bias in Health Care: Development and Validation of a Unified Multistage Framework
Source: JMIR AI. 2026 Aug 14;5:e102146. doi: 10.2196/102146 (PMC13476005; doi:10.2196/102146)
Supplement: Supplementary file 1 [file ai-v5-e102146-s001.docx]

Supplementary Tables: Fresh Reproducibility Reconstruction

*Detecting and Mitigating AI Bias in Healthcare*

Scope: These tables report the operational mappings and results generated by the fresh run (seed 20250117). Source-absent concepts are explicitly marked; they were not imputed as clinical zeros. Bootstrap intervals use 500 replicates overall and 300 replicates for subgroup estimates.

# Supplementary Table S1. Complete 37-concept audit dictionary

| **Concept** | **Type** | **Diabetes source** | **CMS source** | **NHANES source** | **Allowed values** | **Mapping rule** | **Missingness strategy** |
| --- | --- | --- | --- | --- | --- | --- | --- |
| race | Categorical/binary | race | BENE_RACE_CD -> race | RIDRETH1/RIDRETH3 | Source codebook categories or Missing | Direct source mapping or documented derived field; absent sources explicitly masked. | Explicit source-absence/missing indicator; model imputation fitted on training data only. |
| sex | Categorical/binary | gender -> sex | BENE_SEX_IDENT_CD -> sex | RIAGENDR | Source codebook categories or Missing | Direct source mapping or documented derived field; absent sources explicitly masked. | Explicit source-absence/missing indicator; model imputation fitted on training data only. |
| age_group | Categorical/binary | age | age at claim -> band | RIDAGEYR -> bands | 0-17, 18-29, 30-39, 40-49, 50-59, 60-69, 70-79, 80+, Missing | Map source age to prespecified bands. | Explicit source-absence/missing indicator; model imputation fitted on training data only. |
| income_proxy | Numeric/context | payer_code | state_code | INDFMPIR | Finite source value or Missing | Direct source mapping or documented derived field; absent sources explicitly masked. | Explicit source-absence/missing indicator; model imputation fitted on training data only. |
| n_prior_visits | Nonnegative integer/count | number_inpatient + number_outpatient + number_emergency | prior inpatient claims | Source absent | >=0 or Missing | Sum/count only events before index; do not use outcome-window events. | Explicit source-absence/missing indicator; model imputation fitted on training data only. |
| admission_type | Categorical/binary | admission_type_id | Source absent | Source absent | Source codebook categories or Missing | Direct source mapping or documented derived field; absent sources explicitly masked. | Explicit source-absence/missing indicator; model imputation fitted on training data only. |
| admission_source | Categorical/binary | admission_source_id | Source absent | Source absent | Source codebook categories or Missing | Direct source mapping or documented derived field; absent sources explicitly masked. | Explicit source-absence/missing indicator; model imputation fitted on training data only. |
| discharge_disposition | Categorical/binary | discharge_disposition_id | Source absent | Source absent | Source codebook categories or Missing | Direct source mapping or documented derived field; absent sources explicitly masked. | Explicit source-absence/missing indicator; model imputation fitted on training data only. |
| time_in_hospital | Nonnegative integer/count | time_in_hospital | CLM_UTLZTN_DAY_CNT | Source absent | >=0 or Missing | Direct source mapping or documented derived field; absent sources explicitly masked. | Explicit source-absence/missing indicator; model imputation fitted on training data only. |
| num_lab_procedures | Nonnegative integer/count | num_lab_procedures | Source absent | Source absent | >=0 or Missing | Direct source mapping or documented derived field; absent sources explicitly masked. | Explicit source-absence/missing indicator; model imputation fitted on training data only. |
| num_procedures | Nonnegative integer/count | num_procedures | Source absent | Source absent | >=0 or Missing | Direct source mapping or documented derived field; absent sources explicitly masked. | Explicit source-absence/missing indicator; model imputation fitted on training data only. |
| num_medications | Nonnegative integer/count | num_medications | Source absent | Source absent | >=0 or Missing | Direct source mapping or documented derived field; absent sources explicitly masked. | Explicit source-absence/missing indicator; model imputation fitted on training data only. |
| number_outpatient | Nonnegative integer/count | number_outpatient | Source absent | Source absent | >=0 or Missing | Direct source mapping or documented derived field; absent sources explicitly masked. | Explicit source-absence/missing indicator; model imputation fitted on training data only. |
| number_emergency | Nonnegative integer/count | number_emergency | Source absent | Source absent | >=0 or Missing | Direct source mapping or documented derived field; absent sources explicitly masked. | Explicit source-absence/missing indicator; model imputation fitted on training data only. |
| number_inpatient | Nonnegative integer/count | number_inpatient | Source absent | Source absent | >=0 or Missing | Direct source mapping or documented derived field; absent sources explicitly masked. | Explicit source-absence/missing indicator; model imputation fitted on training data only. |
| number_diagnoses | Nonnegative integer/count | number_diagnoses | Source absent | Source absent | >=0 or Missing | Direct source mapping or documented derived field; absent sources explicitly masked. | Explicit source-absence/missing indicator; model imputation fitted on training data only. |
| primary_diagnosis | Categorical/binary | diag_1 | ADMTNG_ICD9_DGNS_CD | Source absent | Valid source ICD-9 code or Missing | Direct source mapping or documented derived field; absent sources explicitly masked. | Explicit source-absence/missing indicator; model imputation fitted on training data only. |
| secondary_diagnosis | Categorical/binary | diag_2 | Source absent | Source absent | Valid source ICD-9 code or Missing | Direct source mapping or documented derived field; absent sources explicitly masked. | Explicit source-absence/missing indicator; model imputation fitted on training data only. |
| tertiary_diagnosis | Categorical/binary | diag_3 | Source absent | Source absent | Valid source ICD-9 code or Missing | Direct source mapping or documented derived field; absent sources explicitly masked. | Explicit source-absence/missing indicator; model imputation fitted on training data only. |
| diagnosis_burden | Nonnegative integer/count | number_diagnoses | diagnosis_count | Source absent | >=0 or Missing | Direct source mapping or documented derived field; absent sources explicitly masked. | Explicit source-absence/missing indicator; model imputation fitted on training data only. |
| hba1c | Categorical/binary | A1Cresult | Source absent | LBXGH | Source categorical A1C result or numeric HbA1c %, by dataset | Direct source mapping or documented derived field; absent sources explicitly masked. | Explicit source-absence/missing indicator; model imputation fitted on training data only. |
| max_glucose | Categorical/binary | max_glu_serum | Source absent | Source absent | Source codebook categories or Missing | Direct source mapping or documented derived field; absent sources explicitly masked. | Explicit source-absence/missing indicator; model imputation fitted on training data only. |
| insulin | Categorical/binary | insulin | Source absent | Source absent | No, Steady, Up, Down, Missing (where available) | Direct source mapping or documented derived field; absent sources explicitly masked. | Explicit source-absence/missing indicator; model imputation fitted on training data only. |
| metformin | Categorical/binary | metformin | Source absent | Source absent | No, Steady, Up, Down, Missing (where available) | Direct source mapping or documented derived field; absent sources explicitly masked. | Explicit source-absence/missing indicator; model imputation fitted on training data only. |
| diabetes_medication | Categorical/binary | diabetesMed | Source absent | Source absent | Source codebook categories or Missing | Direct source mapping or documented derived field; absent sources explicitly masked. | Explicit source-absence/missing indicator; model imputation fitted on training data only. |
| medication_change | Categorical/binary | change | Source absent | Source absent | Source codebook categories or Missing | Direct source mapping or documented derived field; absent sources explicitly masked. | Explicit source-absence/missing indicator; model imputation fitted on training data only. |
| payer_context | Categorical/binary | payer_code | Source absent | Source absent | Source codebook categories or Missing | Direct source mapping or documented derived field; absent sources explicitly masked. | Explicit source-absence/missing indicator; model imputation fitted on training data only. |
| medical_specialty | Categorical/binary | medical_specialty | Source absent | Source absent | Source codebook categories or Missing | Direct source mapping or documented derived field; absent sources explicitly masked. | Explicit source-absence/missing indicator; model imputation fitted on training data only. |
| provider_context | Categorical/binary | Source absent | PRVDR_NUM | Source absent | Source codebook categories or Missing | Direct source mapping or documented derived field; absent sources explicitly masked. | Explicit source-absence/missing indicator; model imputation fitted on training data only. |
| state_context | Categorical/binary | Source absent | state_code | Source absent | Source codebook categories or Missing | Direct source mapping or documented derived field; absent sources explicitly masked. | Explicit source-absence/missing indicator; model imputation fitted on training data only. |
| county_context | Categorical/binary | Source absent | BENE_COUNTY_CD | Source absent | Source codebook categories or Missing | Direct source mapping or documented derived field; absent sources explicitly masked. | Explicit source-absence/missing indicator; model imputation fitted on training data only. |
| claim_payment | Numeric/context | Source absent | CLM_PMT_AMT | Source absent | Finite source value or Missing | Direct source mapping or documented derived field; absent sources explicitly masked. | Explicit source-absence/missing indicator; model imputation fitted on training data only. |
| utilization_days | Nonnegative integer/count | Source absent | CLM_UTLZTN_DAY_CNT | Source absent | >=0 or Missing | Direct source mapping or documented derived field; absent sources explicitly masked. | Explicit source-absence/missing indicator; model imputation fitted on training data only. |
| esrd_indicator | Categorical/binary | Source absent | BENE_ESRD_IND | Source absent | Source codebook categories or Missing | Direct source mapping or documented derived field; absent sources explicitly masked. | Explicit source-absence/missing indicator; model imputation fitted on training data only. |
| comorbidity_burden | Nonnegative integer/count | count nonmissing diag_1:diag_3 | diagnosis_count | Source absent | >=0 or Missing | Count available diagnosis/comorbidity indicators. | Explicit source-absence/missing indicator; model imputation fitted on training data only. |
| temporal_context | Categorical/binary | Source absent | year(admit_date) | NHANES cycle | Source codebook categories or Missing | Direct source mapping or documented derived field; absent sources explicitly masked. | Explicit source-absence/missing indicator; model imputation fitted on training data only. |
| integrity_flag | Categorical/binary | derived: record missingness >=25% | derived: record missingness >=25% | derived: concept missingness >=25% | 0/1 | Derived audit flag; never treated as confirmed error. | Explicit source-absence/missing indicator; model imputation fitted on training data only. |

# Supplementary Table S2. Concept missingness by dataset

| **Concept** | **Diabetes missing n** | **Diabetes %** | **Absent** | **CMS missing n** | **CMS %** | **Absent** | **NHANES missing n** | **NHANES %** | **Absent** |
| --- | --- | --- | --- | --- | --- | --- | --- | --- | --- |
| race | 0 | 0.00% | No | 0 | 0.00% | No | 0 | 0.00% | No |
| sex | 0 | 0.00% | No | 0 | 0.00% | No | 0 | 0.00% | No |
| age_group | 0 | 0.00% | No | 0 | 0.00% | No | 0 | 0.00% | No |
| income_proxy | 40,256 | 39.56% | No | 0 | 0.00% | No | 5,688 | 8.00% | No |
| n_prior_visits | 0 | 0.00% | No | 0 | 0.00% | No | 71,058 | 100.00% | Yes |
| admission_type | 0 | 0.00% | No | 63,996 | 100.00% | Yes | 71,058 | 100.00% | Yes |
| admission_source | 0 | 0.00% | No | 63,996 | 100.00% | Yes | 71,058 | 100.00% | Yes |
| discharge_disposition | 0 | 0.00% | No | 63,996 | 100.00% | Yes | 71,058 | 100.00% | Yes |
| time_in_hospital | 0 | 0.00% | No | 0 | 0.00% | No | 71,058 | 100.00% | Yes |
| num_lab_procedures | 0 | 0.00% | No | 63,996 | 100.00% | Yes | 71,058 | 100.00% | Yes |
| num_procedures | 0 | 0.00% | No | 63,996 | 100.00% | Yes | 71,058 | 100.00% | Yes |
| num_medications | 0 | 0.00% | No | 63,996 | 100.00% | Yes | 71,058 | 100.00% | Yes |
| number_outpatient | 0 | 0.00% | No | 63,996 | 100.00% | Yes | 71,058 | 100.00% | Yes |
| number_emergency | 0 | 0.00% | No | 63,996 | 100.00% | Yes | 71,058 | 100.00% | Yes |
| number_inpatient | 0 | 0.00% | No | 63,996 | 100.00% | Yes | 71,058 | 100.00% | Yes |
| number_diagnoses | 0 | 0.00% | No | 63,996 | 100.00% | Yes | 71,058 | 100.00% | Yes |
| primary_diagnosis | 21 | 0.02% | No | 484 | 0.76% | No | 71,058 | 100.00% | Yes |
| secondary_diagnosis | 358 | 0.35% | No | 63,996 | 100.00% | Yes | 71,058 | 100.00% | Yes |
| tertiary_diagnosis | 1,423 | 1.40% | No | 63,996 | 100.00% | Yes | 71,058 | 100.00% | Yes |
| diagnosis_burden | 0 | 0.00% | No | 0 | 0.00% | No | 71,058 | 100.00% | Yes |
| hba1c | 84,748 | 83.28% | No | 63,996 | 100.00% | Yes | 25,493 | 35.88% | No |
| max_glucose | 96,420 | 94.75% | No | 63,996 | 100.00% | Yes | 71,058 | 100.00% | Yes |
| insulin | 0 | 0.00% | No | 63,996 | 100.00% | Yes | 71,058 | 100.00% | Yes |
| metformin | 0 | 0.00% | No | 63,996 | 100.00% | Yes | 71,058 | 100.00% | Yes |
| diabetes_medication | 0 | 0.00% | No | 63,996 | 100.00% | Yes | 71,058 | 100.00% | Yes |
| medication_change | 0 | 0.00% | No | 63,996 | 100.00% | Yes | 71,058 | 100.00% | Yes |
| payer_context | 40,256 | 39.56% | No | 63,996 | 100.00% | Yes | 71,058 | 100.00% | Yes |
| medical_specialty | 49,949 | 49.08% | No | 63,996 | 100.00% | Yes | 71,058 | 100.00% | Yes |
| provider_context | 101,766 | 100.00% | Yes | 0 | 0.00% | No | 71,058 | 100.00% | Yes |
| state_context | 101,766 | 100.00% | Yes | 0 | 0.00% | No | 71,058 | 100.00% | Yes |
| county_context | 101,766 | 100.00% | Yes | 216 | 0.34% | No | 71,058 | 100.00% | Yes |
| claim_payment | 101,766 | 100.00% | Yes | 0 | 0.00% | No | 71,058 | 100.00% | Yes |
| utilization_days | 101,766 | 100.00% | Yes | 0 | 0.00% | No | 71,058 | 100.00% | Yes |
| esrd_indicator | 101,766 | 100.00% | Yes | 216 | 0.34% | No | 71,058 | 100.00% | Yes |
| comorbidity_burden | 0 | 0.00% | No | 63,996 | 100.00% | Yes | 71,058 | 100.00% | Yes |
| temporal_context | 101,766 | 100.00% | Yes | 0 | 0.00% | No | 0 | 0.00% | No |
| integrity_flag | 0 | 0.00% | No | 0 | 0.00% | No | 0 | 0.00% | No |

# Supplementary Table S3. Primary test-set subgroup sensitivity analysis, performance, and calibration

| **Model** | **Group** | **Level** | **n** | **Positive** | **Prev.** | **Metric** | **Estimate** | **95% CI low** | **95% CI high** | **Boot n** |
| --- | --- | --- | --- | --- | --- | --- | --- | --- | --- | --- |
| M2 | race | AfricanAmerican | 3,927 | 441 | 11.23% | auc | 0.640 | 0.611 | 0.666 | 300 |
| M2 | race | AfricanAmerican | 3,927 | 441 | 11.23% | sensitivity | 0.526 | 0.478 | 0.575 | 300 |
| M2 | race | AfricanAmerican | 3,927 | 441 | 11.23% | specificity | 0.671 | 0.654 | 0.686 | 300 |
| M2 | race | AfricanAmerican | 3,927 | 441 | 11.23% | ppv | 0.168 | 0.153 | 0.183 | 300 |
| M2 | race | AfricanAmerican | 3,927 | 441 | 11.23% | npv | 0.918 | 0.910 | 0.926 | 300 |
| M2 | race | AfricanAmerican | 3,927 | 441 | 11.23% | brier | 0.231 | 0.229 | 0.233 | 300 |
| M2 | race | AfricanAmerican | 3,927 | 441 | 11.23% | calibration_intercept | -1.999 | -2.020 | -1.985 | 300 |
| M2 | race | AfricanAmerican | 3,927 | 441 | 11.23% | calibration_slope | 1.806 | 1.468 | 2.175 | 300 |
| M2 | race | AfricanAmerican | 3,927 | 441 | 11.23% | ece_10 | 0.366 | 0.364 | 0.368 | 300 |
| M3 | race | AfricanAmerican | 3,927 | 441 | 11.23% | auc | 0.635 | 0.610 | 0.661 | 300 |
| M3 | race | AfricanAmerican | 3,927 | 441 | 11.23% | sensitivity | 0.390 | 0.349 | 0.432 | 300 |
| M3 | race | AfricanAmerican | 3,927 | 441 | 11.23% | specificity | 0.772 | 0.756 | 0.786 | 300 |
| M3 | race | AfricanAmerican | 3,927 | 441 | 11.23% | ppv | 0.178 | 0.160 | 0.197 | 300 |
| M3 | race | AfricanAmerican | 3,927 | 441 | 11.23% | npv | 0.909 | 0.903 | 0.915 | 300 |
| M3 | race | AfricanAmerican | 3,927 | 441 | 11.23% | brier | 0.213 | 0.211 | 0.214 | 300 |
| M3 | race | AfricanAmerican | 3,927 | 441 | 11.23% | calibration_intercept | -1.822 | -1.858 | -1.785 | 300 |
| M3 | race | AfricanAmerican | 3,927 | 441 | 11.23% | calibration_slope | 1.652 | 1.343 | 1.989 | 300 |
| M3 | race | AfricanAmerican | 3,927 | 441 | 11.23% | ece_10 | 0.338 | 0.336 | 0.341 | 300 |
| M2 | race | Caucasian | 15,113 | 1,717 | 11.36% | auc | 0.646 | 0.632 | 0.659 | 300 |
| M2 | race | Caucasian | 15,113 | 1,717 | 11.36% | sensitivity | 0.545 | 0.520 | 0.565 | 300 |
| M2 | race | Caucasian | 15,113 | 1,717 | 11.36% | specificity | 0.657 | 0.649 | 0.664 | 300 |
| M2 | race | Caucasian | 15,113 | 1,717 | 11.36% | ppv | 0.169 | 0.161 | 0.175 | 300 |
| M2 | race | Caucasian | 15,113 | 1,717 | 11.36% | npv | 0.918 | 0.914 | 0.922 | 300 |
| M2 | race | Caucasian | 15,113 | 1,717 | 11.36% | brier | 0.232 | 0.231 | 0.233 | 300 |
| M2 | race | Caucasian | 15,113 | 1,717 | 11.36% | calibration_intercept | -1.988 | -1.997 | -1.981 | 300 |
| M2 | race | Caucasian | 15,113 | 1,717 | 11.36% | calibration_slope | 2.080 | 1.881 | 2.277 | 300 |
| M2 | race | Caucasian | 15,113 | 1,717 | 11.36% | ece_10 | 0.366 | 0.365 | 0.367 | 300 |
| M3 | race | Caucasian | 15,113 | 1,717 | 11.36% | auc | 0.636 | 0.623 | 0.651 | 300 |
| M3 | race | Caucasian | 15,113 | 1,717 | 11.36% | sensitivity | 0.393 | 0.372 | 0.419 | 300 |
| M3 | race | Caucasian | 15,113 | 1,717 | 11.36% | specificity | 0.778 | 0.772 | 0.785 | 300 |
| M3 | race | Caucasian | 15,113 | 1,717 | 11.36% | ppv | 0.185 | 0.175 | 0.197 | 300 |
| M3 | race | Caucasian | 15,113 | 1,717 | 11.36% | npv | 0.909 | 0.906 | 0.913 | 300 |
| M3 | race | Caucasian | 15,113 | 1,717 | 11.36% | brier | 0.214 | 0.213 | 0.215 | 300 |
| M3 | race | Caucasian | 15,113 | 1,717 | 11.36% | calibration_intercept | -1.798 | -1.817 | -1.777 | 300 |
| M3 | race | Caucasian | 15,113 | 1,717 | 11.36% | calibration_slope | 1.877 | 1.671 | 2.100 | 300 |
| M3 | race | Caucasian | 15,113 | 1,717 | 11.36% | ece_10 | 0.340 | 0.339 | 0.341 | 300 |
| M2 | race | Hispanic | 368 | 28 | 7.61% | auc | 0.782 | 0.706 | 0.854 | 300 |
| M2 | race | Hispanic | 368 | 28 | 7.61% | sensitivity | 0.607 | 0.429 | 0.786 | 300 |
| M2 | race | Hispanic | 368 | 28 | 7.61% | specificity | 0.741 | 0.700 | 0.787 | 300 |
| M2 | race | Hispanic | 368 | 28 | 7.61% | ppv | 0.162 | 0.116 | 0.213 | 300 |
| M2 | race | Hispanic | 368 | 28 | 7.61% | npv | 0.958 | 0.940 | 0.976 | 300 |
| M2 | race | Hispanic | 368 | 28 | 7.61% | brier | 0.215 | 0.208 | 0.221 | 300 |
| M2 | race | Hispanic | 368 | 28 | 7.61% | calibration_intercept | -2.389 | -2.583 | -2.257 | 300 |
| M2 | race | Hispanic | 368 | 28 | 7.61% | calibration_slope | 3.844 | 2.596 | 5.330 | 300 |
| M2 | race | Hispanic | 368 | 28 | 7.61% | ece_10 | 0.387 | 0.380 | 0.395 | 300 |
| M3 | race | Hispanic | 368 | 28 | 7.61% | auc | 0.796 | 0.733 | 0.861 | 300 |
| M3 | race | Hispanic | 368 | 28 | 7.61% | sensitivity | 0.429 | 0.250 | 0.607 | 300 |
| M3 | race | Hispanic | 368 | 28 | 7.61% | specificity | 0.835 | 0.800 | 0.871 | 300 |
| M3 | race | Hispanic | 368 | 28 | 7.61% | ppv | 0.176 | 0.107 | 0.250 | 300 |
| M3 | race | Hispanic | 368 | 28 | 7.61% | npv | 0.947 | 0.931 | 0.963 | 300 |
| M3 | race | Hispanic | 368 | 28 | 7.61% | brier | 0.193 | 0.187 | 0.199 | 300 |
| M3 | race | Hispanic | 368 | 28 | 7.61% | calibration_intercept | -1.983 | -2.124 | -1.846 | 300 |
| M3 | race | Hispanic | 368 | 28 | 7.61% | calibration_slope | 3.904 | 2.760 | 5.440 | 300 |
| M3 | race | Hispanic | 368 | 28 | 7.61% | ece_10 | 0.359 | 0.352 | 0.367 | 300 |
| M2 | race | Missing | 431 | 33 | 7.66% | auc | 0.637 | 0.548 | 0.725 | 300 |
| M2 | race | Missing | 431 | 33 | 7.66% | sensitivity | 0.333 | 0.152 | 0.485 | 300 |
| M2 | race | Missing | 431 | 33 | 7.66% | specificity | 0.751 | 0.710 | 0.794 | 300 |
| M2 | race | Missing | 431 | 33 | 7.66% | ppv | 0.100 | 0.050 | 0.147 | 300 |
| M2 | race | Missing | 431 | 33 | 7.66% | npv | 0.931 | 0.914 | 0.948 | 300 |
| M2 | race | Missing | 431 | 33 | 7.66% | brier | 0.216 | 0.211 | 0.222 | 300 |
| M2 | race | Missing | 431 | 33 | 7.66% | calibration_intercept | -2.265 | -2.414 | -2.161 | 300 |
| M2 | race | Missing | 431 | 33 | 7.66% | calibration_slope | 1.819 | 0.480 | 3.213 | 300 |
| M2 | race | Missing | 431 | 33 | 7.66% | ece_10 | 0.382 | 0.376 | 0.387 | 300 |
| M3 | race | Missing | 431 | 33 | 7.66% | auc | 0.609 | 0.509 | 0.697 | 300 |
| M3 | race | Missing | 431 | 33 | 7.66% | sensitivity | 0.182 | 0.061 | 0.319 | 300 |
| M3 | race | Missing | 431 | 33 | 7.66% | specificity | 0.877 | 0.843 | 0.911 | 300 |
| M3 | race | Missing | 431 | 33 | 7.66% | ppv | 0.109 | 0.030 | 0.193 | 300 |
| M3 | race | Missing | 431 | 33 | 7.66% | npv | 0.928 | 0.917 | 0.940 | 300 |
| M3 | race | Missing | 431 | 33 | 7.66% | brier | 0.197 | 0.192 | 0.203 | 300 |
| M3 | race | Missing | 431 | 33 | 7.66% | calibration_intercept | -2.131 | -2.439 | -1.898 | 300 |
| M3 | race | Missing | 431 | 33 | 7.66% | calibration_slope | 1.479 | 0.182 | 2.818 | 300 |
| M3 | race | Missing | 431 | 33 | 7.66% | ece_10 | 0.354 | 0.349 | 0.360 | 300 |
| M2 | race | Other | 258 | 27 | 10.47% | auc | 0.631 | 0.515 | 0.747 | 300 |
| M2 | race | Other | 258 | 27 | 10.47% | sensitivity | 0.481 | 0.333 | 0.667 | 300 |
| M2 | race | Other | 258 | 27 | 10.47% | specificity | 0.749 | 0.686 | 0.807 | 300 |
| M2 | race | Other | 258 | 27 | 10.47% | ppv | 0.183 | 0.125 | 0.258 | 300 |
| M2 | race | Other | 258 | 27 | 10.47% | npv | 0.925 | 0.903 | 0.950 | 300 |
| M2 | race | Other | 258 | 27 | 10.47% | brier | 0.221 | 0.213 | 0.228 | 300 |
| M2 | race | Other | 258 | 27 | 10.47% | calibration_intercept | -1.949 | -2.120 | -1.872 | 300 |
| M2 | race | Other | 258 | 27 | 10.47% | calibration_slope | 2.233 | 0.296 | 4.237 | 300 |
| M2 | race | Other | 258 | 27 | 10.47% | ece_10 | 0.361 | 0.353 | 0.374 | 300 |
| M3 | race | Other | 258 | 27 | 10.47% | auc | 0.631 | 0.503 | 0.751 | 300 |
| M3 | race | Other | 258 | 27 | 10.47% | sensitivity | 0.444 | 0.259 | 0.630 | 300 |
| M3 | race | Other | 258 | 27 | 10.47% | specificity | 0.870 | 0.827 | 0.918 | 300 |
| M3 | race | Other | 258 | 27 | 10.47% | ppv | 0.286 | 0.171 | 0.395 | 300 |
| M3 | race | Other | 258 | 27 | 10.47% | npv | 0.931 | 0.908 | 0.953 | 300 |
| M3 | race | Other | 258 | 27 | 10.47% | brier | 0.200 | 0.193 | 0.208 | 300 |
| M3 | race | Other | 258 | 27 | 10.47% | calibration_intercept | -1.732 | -2.117 | -1.491 | 300 |
| M3 | race | Other | 258 | 27 | 10.47% | calibration_slope | 1.950 | 0.114 | 3.894 | 300 |
| M3 | race | Other | 258 | 27 | 10.47% | ece_10 | 0.333 | 0.324 | 0.348 | 300 |
| M2 | sex | Female | 10,909 | 1,181 | 10.83% | auc | 0.649 | 0.633 | 0.664 | 300 |
| M2 | sex | Female | 10,909 | 1,181 | 10.83% | sensitivity | 0.550 | 0.522 | 0.577 | 300 |
| M2 | sex | Female | 10,909 | 1,181 | 10.83% | specificity | 0.659 | 0.650 | 0.666 | 300 |
| M2 | sex | Female | 10,909 | 1,181 | 10.83% | ppv | 0.163 | 0.156 | 0.172 | 300 |
| M2 | sex | Female | 10,909 | 1,181 | 10.83% | npv | 0.923 | 0.919 | 0.928 | 300 |
| M2 | sex | Female | 10,909 | 1,181 | 10.83% | brier | 0.232 | 0.231 | 0.233 | 300 |
| M2 | sex | Female | 10,909 | 1,181 | 10.83% | calibration_intercept | -2.043 | -2.054 | -2.033 | 300 |
| M2 | sex | Female | 10,909 | 1,181 | 10.83% | calibration_slope | 2.080 | 1.836 | 2.311 | 300 |
| M2 | sex | Female | 10,909 | 1,181 | 10.83% | ece_10 | 0.371 | 0.370 | 0.372 | 300 |
| M3 | sex | Female | 10,909 | 1,181 | 10.83% | auc | 0.640 | 0.626 | 0.656 | 300 |
| M3 | sex | Female | 10,909 | 1,181 | 10.83% | sensitivity | 0.386 | 0.359 | 0.415 | 300 |
| M3 | sex | Female | 10,909 | 1,181 | 10.83% | specificity | 0.772 | 0.763 | 0.780 | 300 |
| M3 | sex | Female | 10,909 | 1,181 | 10.83% | ppv | 0.170 | 0.160 | 0.181 | 300 |
| M3 | sex | Female | 10,909 | 1,181 | 10.83% | npv | 0.912 | 0.908 | 0.916 | 300 |
| M3 | sex | Female | 10,909 | 1,181 | 10.83% | brier | 0.214 | 0.213 | 0.215 | 300 |
| M3 | sex | Female | 10,909 | 1,181 | 10.83% | calibration_intercept | -1.855 | -1.877 | -1.834 | 300 |
| M3 | sex | Female | 10,909 | 1,181 | 10.83% | calibration_slope | 1.871 | 1.674 | 2.103 | 300 |
| M3 | sex | Female | 10,909 | 1,181 | 10.83% | ece_10 | 0.346 | 0.345 | 0.347 | 300 |
| M2 | sex | Male | 9,292 | 1,073 | 11.55% | auc | 0.647 | 0.629 | 0.665 | 300 |
| M2 | sex | Male | 9,292 | 1,073 | 11.55% | sensitivity | 0.527 | 0.495 | 0.557 | 300 |
| M2 | sex | Male | 9,292 | 1,073 | 11.55% | specificity | 0.674 | 0.663 | 0.684 | 300 |
| M2 | sex | Male | 9,292 | 1,073 | 11.55% | ppv | 0.174 | 0.164 | 0.183 | 300 |
| M2 | sex | Male | 9,292 | 1,073 | 11.55% | npv | 0.916 | 0.911 | 0.921 | 300 |
| M2 | sex | Male | 9,292 | 1,073 | 11.55% | brier | 0.230 | 0.229 | 0.231 | 300 |
| M2 | sex | Male | 9,292 | 1,073 | 11.55% | calibration_intercept | -1.954 | -1.966 | -1.941 | 300 |
| M2 | sex | Male | 9,292 | 1,073 | 11.55% | calibration_slope | 2.052 | 1.807 | 2.328 | 300 |
| M2 | sex | Male | 9,292 | 1,073 | 11.55% | ece_10 | 0.362 | 0.360 | 0.363 | 300 |
| M3 | sex | Male | 9,292 | 1,073 | 11.55% | auc | 0.639 | 0.622 | 0.657 | 300 |
| M3 | sex | Male | 9,292 | 1,073 | 11.55% | sensitivity | 0.397 | 0.370 | 0.424 | 300 |
| M3 | sex | Male | 9,292 | 1,073 | 11.55% | specificity | 0.794 | 0.785 | 0.803 | 300 |
| M3 | sex | Male | 9,292 | 1,073 | 11.55% | ppv | 0.201 | 0.189 | 0.214 | 300 |
| M3 | sex | Male | 9,292 | 1,073 | 11.55% | npv | 0.910 | 0.906 | 0.914 | 300 |
| M3 | sex | Male | 9,292 | 1,073 | 11.55% | brier | 0.211 | 0.210 | 0.213 | 300 |
| M3 | sex | Male | 9,292 | 1,073 | 11.55% | calibration_intercept | -1.753 | -1.780 | -1.728 | 300 |
| M3 | sex | Male | 9,292 | 1,073 | 11.55% | calibration_slope | 1.876 | 1.644 | 2.099 | 300 |
| M3 | sex | Male | 9,292 | 1,073 | 11.55% | ece_10 | 0.334 | 0.333 | 0.336 | 300 |

# Supplementary Table S4. Dataset and split flow

| **Dataset** | **Split/role** | **Records** | **Unique individuals** | **Positive outcomes** | **Prevalence** |
| --- | --- | --- | --- | --- | --- |
| diabetes | all_eligible | 101,766 | 71,518 | 11,357 | 11.16% |
| diabetes | train | 81,563 | 57,214 | 9,103 | 11.16% |
| diabetes | test | 20,203 | 14,304 | 2,254 | 11.16% |
| cms | all_eligible | 63,996 | 37,702 | 6,107 | 9.54% |
| cms | train | 51,195 | 30,161 | 4,875 | 9.52% |
| cms | test | 12,801 | 7,541 | 1,232 | 9.62% |
| nhanes | stage_level_all | 71,058 | 71,058 | 5,980 | 8.42% |

# Supplementary Table S5. Overall performance and fairness uncertainty

| **Dataset** | **Model** | **Metric** | **Estimate** | **95% CI low** | **95% CI high** | **Boot n** |
| --- | --- | --- | --- | --- | --- | --- |
| test | M2 | auc | 0.648 | 0.637 | 0.660 | 500 |
| test | M2 | macro_f1 | 0.514 | 0.508 | 0.521 | 500 |
| test | M2 | brier | 0.231 | 0.230 | 0.232 | 500 |
| test | M2 | sensitivity | 0.539 | 0.518 | 0.559 | 500 |
| test | M2 | specificity | 0.666 | 0.659 | 0.672 | 500 |
| test | M2 | ppv | 0.168 | 0.162 | 0.174 | 500 |
| test | M2 | npv | 0.920 | 0.916 | 0.923 | 500 |
| test | M2 | dpd | 0.206 | 0.147 | 0.268 | 500 |
| test | M2 | equalized_odds_difference | 0.417 | 0.230 | 0.758 | 500 |
| test | M2 | eor | 0.444 | 0.222 | 0.685 | 500 |
| test | M2 | calibration_intercept | -2.001 | -2.010 | -1.993 | 500 |
| test | M2 | calibration_slope | 2.063 | 1.914 | 2.215 | 500 |
| test | M2 | ece_10 | 0.366 | 0.366 | 0.367 | 500 |
| test | M3 | auc | 0.640 | 0.628 | 0.652 | 500 |
| test | M3 | macro_f1 | 0.546 | 0.539 | 0.553 | 500 |
| test | M3 | brier | 0.213 | 0.212 | 0.214 | 500 |
| test | M3 | sensitivity | 0.391 | 0.372 | 0.411 | 500 |
| test | M3 | specificity | 0.782 | 0.776 | 0.788 | 500 |
| test | M3 | ppv | 0.184 | 0.175 | 0.193 | 500 |
| test | M3 | npv | 0.911 | 0.908 | 0.914 | 500 |
| test | M3 | dpd | 0.124 | 0.101 | 0.185 | 500 |
| test | M3 | equalized_odds_difference | 0.443 | 0.227 | 0.784 | 500 |
| test | M3 | eor | 0.291 | 0.097 | 0.545 | 500 |
| test | M3 | calibration_intercept | -1.809 | -1.824 | -1.791 | 500 |
| test | M3 | calibration_slope | 1.874 | 1.718 | 2.031 | 500 |
| test | M3 | ece_10 | 0.340 | 0.340 | 0.341 | 500 |
| cms | M2 | auc | 0.798 | 0.786 | 0.808 | 500 |
| cms | M2 | macro_f1 | 0.575 | 0.568 | 0.583 | 500 |
| cms | M2 | brier | 0.187 | 0.185 | 0.189 | 500 |
| cms | M2 | sensitivity | 0.809 | 0.789 | 0.830 | 500 |
| cms | M2 | specificity | 0.691 | 0.682 | 0.699 | 500 |
| cms | M2 | ppv | 0.218 | 0.211 | 0.224 | 500 |
| cms | M2 | npv | 0.971 | 0.968 | 0.975 | 500 |
| cms | M2 | dpd | 0.693 | 0.655 | 0.733 | 500 |
| cms | M2 | equalized_odds_difference | 0.732 | 0.697 | 0.777 | 500 |
| cms | M2 | eor | 0.656 | 0.500 | 0.778 | 500 |
| cms | M2 | calibration_intercept | -2.151 | -2.184 | -2.120 | 500 |
| cms | M2 | calibration_slope | 2.052 | 1.930 | 2.174 | 500 |
| cms | M2 | ece_10 | 0.325 | 0.323 | 0.328 | 500 |
| cms | M3 | auc | 0.796 | 0.785 | 0.806 | 500 |
| cms | M3 | macro_f1 | 0.583 | 0.575 | 0.591 | 500 |
| cms | M3 | brier | 0.183 | 0.181 | 0.185 | 500 |
| cms | M3 | sensitivity | 0.772 | 0.749 | 0.795 | 500 |
| cms | M3 | specificity | 0.712 | 0.704 | 0.721 | 500 |
| cms | M3 | ppv | 0.222 | 0.215 | 0.230 | 500 |
| cms | M3 | npv | 0.967 | 0.964 | 0.970 | 500 |
| cms | M3 | dpd | 0.730 | 0.697 | 0.771 | 500 |
| cms | M3 | equalized_odds_difference | 0.768 | 0.734 | 0.809 | 500 |
| cms | M3 | eor | 0.625 | 0.438 | 0.742 | 500 |
| cms | M3 | calibration_intercept | -2.084 | -2.112 | -2.058 | 500 |
| cms | M3 | calibration_slope | 2.048 | 1.933 | 2.167 | 500 |
| cms | M3 | ece_10 | 0.320 | 0.317 | 0.322 | 500 |

# Supplementary Table S6. Explicit three-arm MoE comparison and paired contrasts versus M3

| **Metric** | **MoE-M3 difference** | **95% CI low** | **95% CI high** | **Boot n** |
| --- | --- | --- | --- | --- |
| auc | 0.0078 | 0.0034 | 0.0122 | 500 |
| brier | 0.0177 | 0.0175 | 0.0180 | 500 |
| dpd | 0.0708 | 0.0154 | 0.1172 | 500 |
| eor | 0.1348 | -0.1225 | 0.4111 | 500 |
| macro_f1 | -0.0316 | -0.0372 | -0.0264 | 500 |

Sensitivity-analysis results: At threshold 0.50, M2 sensitivity ranged from 0.333 to 0.607 across race strata and was 0.550 in females and 0.527 in males; M3 sensitivity ranged from 0.182 to 0.444 across race strata and was 0.386 in females and 0.397 in males. The full stratum-specific estimates and 95% confidence intervals are reported in Table S3. In the three-arm aggregate comparison, M2/M3/MoE estimates were AUC 0.648/0.640/0.648, Macro F1 0.514/0.546/0.514, Brier 0.231/0.213/0.231, DPD 0.206/0.124/0.205, and EOR 0.444/0.291/0.444; paired MoE-minus-M3 intervals are reported in Table S6.

## Supplementary Table S7. Pseudocode for the Seven Auditable Procedures

## Moved from the main manuscript Methods section to keep the main text focused on the empirical pipeline executed in the fresh reconstruction. Algorithm numbering (1-7) is unchanged from the main text.

## Pseudocode

**Algorithm 1. Representation-bias audit and weighting.**

1. Input: H_s, sensitive attribute a, candidate target distribution P_target
2. Audit P_target for geographic, temporal, and coverage mismatch
3. If mismatch is detected: blend P_target with internal empirical prior
4. Compute w(a) = P_target(a) / P_obs(a)
5. Clip weights at selected percentile from nested cross-validation
6. Return weighted dataset H_repr

**Algorithm 2. Proxy-audit sensitivity analysis (fresh reconstruction).**

**Procedure**

1. Stratify M3 results by income_proxy availability and by n_prior_visits equal to zero versus positive.

2. Report AUC, Macro F1, Brier, DPD, EOR, and calibration; do not claim proxy residualization.

**Algorithm 3. Cross-source audit dictionary and missingness mapping.**

**Procedure**

1. Map each source into the 37 audit concepts listed in Supplementary Table S1.

2. Mark concepts absent from a source explicitly and compute missingness percentages.

3. Use the dictionary for audit comparability; train predictive models within source-specific schemas.

**Algorithm 4. Operational integrity-flag audit.**

**Procedure**

1. Compute record missingness rate and n_prior_visits distribution.

2. Flag records with missingness >=25% or n_prior_visits above the 99th percentile.

3. Retain all flagged records for exploratory routing.

**Algorithm 5. Preparation of main and quality experts.**

**Procedure**

1. Use M2 as the main expert and M3 as the missingness-aware quality expert.

2. Do not train demographic-specific or temporal experts in the fresh reconstruction.

**Algorithm 6. Fixed rule-gated exploratory inference.**

**Procedure**

1. If integrity flag=1, use M3 score; otherwise use M2 score.

2. Evaluate the fixed gate against M2 and M3 with paired bootstrap contrasts.

3. Do not use test labels or demographic labels in routing.

**Algorithm 7. Prespecified temporal audit (not empirically estimable in the fresh Diabetes run).**

**Procedure**

1. Requires reliable encounter time and site identifiers.

2. Not estimable for Diabetes 130-US in the fresh run; report as unevaluated rather than imputing time.
